# Shorter Ice Duration and Changing Phenology Influence Under‐Ice Lake Temperature Dynamics

**DOI:** 10.1029/2024JG008382

**Published:** 2024-11-23

**Authors:** Isabella A. Oleksy, David C. Richardson

**Affiliations:** ^1^ Institute of Arctic and Alpine Research University of Colorado Boulder CO USA; ^2^ Department of Ecology and Evolutionary Biology University of Colorado Boulder Boulder CO USA; ^3^ Biology Department SUNY New Paltz New Paltz NY USA

**Keywords:** phenology, lake ice, variability, climate change, long‐term trends, trend analysis

## Abstract

Temperate lakes worldwide are losing ice cover but the implications for under‐ice thermal dynamics are poorly constrained. Using a 92‐year record of ice phenology from a temperate and historically dimictic lake, we examined trends, variability, and drivers of ice phenology and under‐ice temperatures. The onset of ice formation decreased by 23 days century^−1^, which can be largely attributed to warming air temperatures. Ice‐off date has become substantially more variable with spring air temperatures and cumulative February through April snowfall explaining over 80% of the variation in timing. As a result of changing ice phenology, total ice duration contracted by a month and more than doubled in interannual variability. Using weekly under‐ice temperature profiles for the most recent 36 years, we found that shorter ice duration decreased winter inverse stratification and was associated with an extended spring mixing period. We illustrate the limitations of relying on discrete ice clearance dates in our assumptions around under‐ice thermal dynamics by presenting high‐frequency under‐ice observations in two recent winters: one with intermittent ice cover and a year with slow spring ice clearance.

## Key Points

- Ice duration and ice formation are getting shorter or occurring later, and both are increasing in interannual variability
- Ice break up is not trending earlier, but interannual variability is increasing
- This dimictic lake is at risk of transitioning toward monomixis in the near future

## 1 Introduction

Ice cover is one of the longest‐measured indicators of climate change in lake ecosystems (Magnuson et al., 2000; Robertson et al., 1992). Widespread changes in lake ice phenology are occurring around the world (Sharma et al., 2019). Seasonally ice‐covered lakes, particularly those in the zone in North America and Eurasia with maximum winter temperatures around 0°C, are projected to transition toward either intermittent or complete loss of ice cover due to global anthropogenic climate change (Woolway, Denfeld, et al., 2022; Woolway, Sharma, & Smol, 2022). Lake ice and the timing of its formation and clearance affect many lake ecosystem properties such as oxygen dynamics, the timing of thermal stratification, and lake productivity (Adrian et al., 1999; Jansen et al., 2021; Prowse et al., 2011). Understanding under‐ice thermal dynamics is crucial to further comprehending the impacts of changing ice phenology on lake ecosystems.

Two distinct phases of lake thermal dynamics under ice have been currently identified and are dependent on lake morphometry and local climatic conditions (Kirillin et al., 2012). The first phase is characterized by the release of heat from the sediments that have accumulated in the ice‐free season. The second phase is typically characterized by inverse stratification, although it is important to note that some exceptions exist, such as in shallow lakes that are cold and fully mixed at ice‐onset (cryomictic; Yang et al., 2021). Modeling exercises predict that under several representative concentration pathways (RCPs), we can expect a substantial shortening of inverse stratification duration in ice‐covered lakes (Woolway, Denfeld, et al., 2022).

Predicting how ongoing changes in lake ice phenology will alter annual, whole‐lake ecosystem function first requires an understanding of the drivers of trends and variability around those trends (Wilkinson et al., 2020). Although lake ice phenology records are common, observations of multi‐decadal under‐ice thermal properties are extremely rare (e.g., Sharma et al., 2022). Under‐ice temperatures are critical in contextualizing the physical, chemical, and biological implications of increasingly shorter and potentially variable ice phenology. To that end, we analyzed a 90‐year record of both ice‐on and ice‐off observations from a small lake in Shawangunk Mountains, New York, USA. We combined the long‐term ice phenology record with weekly under‐ice thermal profiles from the most recent 36 years to ask the following questions. First, are ice phenology and variability changing? Second, what local meteorological and global scale climatic variables explain variability in lake ice phenology? Third, how does ice phenology affect underwater winter stratification, temperature, and mixing dynamics? Lastly, we used high‐frequency temperature profiles from two winters (2016–2017 and 2017–2018) to look at several metrics of inverse stratification around the timing of ice formation and compared these observations with visual observations of ice formation (“ice on”).

We expected to find long‐term trends in all aspects of ice phenology (ice‐on, ice‐off, and ice duration). We hypothesized that the timing of ice formation would be primarily controlled by minimum air temperatures in the late fall and early winter, whereas ice‐off would be controlled by a combination of spring air temperatures and cumulative precipitation as snow. We tested the hypothesis that a combination of long‐term global climate anomalies and large‐scale atmospheric circulation patterns (teleconnections) would explain this interannual variability in ice duration (Beyene & Jain, 2015). Finally, we expected that the timing of ice‐on and ice‐off (and thus ice duration) would affect the under‐ice heat content and that the length of ice duration would be related to the strength of inverse stratification.

## 2 Methods

### 2.1 Site Description and Data Collection

Mohonk Lake (41.766°N, −74.158°W, 379 m elevation above sea level) is a small (6.9 ha), deep (18.5 m maximum depth), dimictic, oligo‐mesotrophic lake located on the Shawangunk Ridge, New York State, USA (Oleksy & Richardson, 2021; Richardson et al., 2018). The lake occupies a small glacier‐formed depression in a watershed of 17.3 ha and a drainage ratio of 2.5 (Richardson et al., 2018). Ice‐on day was recorded as the first day with 100% ice coverage; ice‐off day was recorded as the first day with 0% ice cover from 1932 to 2023. In the 2018–2019 winter, there was a gap with ice melt between 23 December 2018 and 11 January 2019. Similarly, in the 2022–2023 winter, there were three periods of ice cover with complete ice melt between 22 December 2022 to 05 January 2023, 02 February 2023 to 18 February 2023, and 25 February 2023 to 23 March 2023. To be consistent with past records, we used the first observation of ice‐on and the last observation of ice‐off for these analyses, but, in our calculations of total ice cover (“ice duration”), we subtracted out the gaps in winters with intermittent ice cover. Ice phenology was recorded in a consistent manner over the entire 92‐year data record with only 7 years missing ice‐on data (all before water year 1954) and 1 year of missing ice‐off record in 1966. Over the entire ice record, maximum, minimum, and average daily air temperature (°C) and snow and precipitation (mm) were measured at a local National Weather Service station (Network ID: GHCND:USC00305426). From 1985 to 2023, profiles of lake temperature were taken weekly at 1 m increments through a dock with a water depth of 13 m (Oleksy & Richardson, 2021). Temperatures were linearly interpolated to daily timescales except if there were more than 14‐day gaps in records. From November 2016 to May 2018, high‐frequency (15‐min) temperature data were collected using NexSens T‐Node temperature loggers at 1‐m intervals from the surface to 9 m deep encompassing two winters (2016–2017 and 2017–2018). Because of power issues and sensor failures, we are missing data at the end of the 2017 winter and from 19 January 2018 to 07 February 2018 and from 02 April 2018 to 12 April 2018. We matched that data with a high‐frequency (1‐hr) meteorology station <5 km from Mohonk Lake (Brotzge et al., 2020) that were linearly interpolated to 15‐min intervals to match the lake thermal data.

### 2.2 Ice Phenology Trends Analysis

All statistical analyses and data visualizations were conducted in R version 4.2.1 (R Core Team, 2022). For the three ice phenology metrics, we calculated Theil‐Sen’s slopes using formulas from both *zyp* and *trend* packages (Bronaugh & Consortium, 2019; Pohlert, 2020). To test if a trend was significant, we used the Mann‐Kendall rank‐based *z*‐score and compared the *p*‐value from that *z*‐score to *α* = 0.05.

To test whether variability of ice is increasing, we used two techniques. First, we calculated the standard deviation (sd) of all possible sequential windows from 4 up to 30 years if >95% of the years had ice phenology data (Figures S1–S4 in Supporting Information S1). For each series across all sequential windows, we calculated Theil‐Sen’s slopes and intercepts to determine if sds were changing (Figures S1–S4 in Supporting Information S1). We averaged all slopes for each sequential window and then compared the averaged slopes to zero using one‐sample *t*‐tests. Second, for visualization, we calculated Bollinger Bands, which are one standard deviation above and below a simple moving average and can indicate volatility of a time series (Bollinger, 1992).

### 2.3 Predictors of Ice Phenology

For predictors of ice phenology, we calculated the cumulative sum of rain or snow and air temperature data at one‐, two‐, and 3‐month intervals for October–December (ice‐on) and February–April (ice‐off). We further calculated fall and spring isotherm dates as the day when a moving average of air temperature crossed a temperature threshold (sensu Higgins et al., 2021). We calculated the isotherm date with a factorial design using a range of daily air temperature metrics (minimum, mean, and maximum), lengths of time (1–30 days), and temperature thresholds (0–5°C) for 540 unique combinations. We selected the isotherm variable that maximized *R* ^2^ for either ice‐on or ice‐off. The annual date that the daily 17‐day maximum observed air temperature crossed below 0°C (hereafter “fall 0°C isotherm date”) was most strongly correlated with ice‐on, and the annual date that the daily 29‐day mean observed air temperature crossed above 4°C (hereafter “spring 4°C isotherm date”) was most strongly correlated with ice‐off.

We modeled ice‐on and ice‐off using generalized additive models (GAMs) consisting of terms that accounted for interannual variability across each time series using a gamma family with a logistic link function (Hastie & Tibshirani, 1987; Wood, 2017). GAMs are effective at capturing nonlinear relationships between environmental variables and response variables‐in this case ice on, off, or duration. We built candidate models based on the top 10 climatic variables that were most highly correlated with either ice‐on or ice‐off while minimizing collinearity (Figure S5 in Supporting Information S1). We fit several GAMs for each ice phenological variable (Table S1 in Supporting Information S1) and ultimately selected the models that had the lower AIC and maximized deviance explained (Table S2 in Supporting Information S1). We constructed GAMs using the *mgcv* package (version 1.8–40; Wood, 2019), visualized all results with the *ggplot2* package (Wickham, 2016), and arranged the plots with *patchwork* package (Pedersen, 2022).

To examine the effects of broader‐scale meteorological factors, we built GAMs including global temperature and teleconnections as potential drivers of ice duration. We obtained global annual temperature anomalies averaged over land and ocean from the National Oceanic and Atmospheric Administration (NOAA, 2020). We also considered monthly North Atlantic Oscillation (NAO) and multivariate El Niño/Southern Oscillation (ENSO) indices as predictors of ice phenology, which we downloaded from the National Weather Service Climate Prediction Center (National Weather Service, 2020) and National Oceanic and Atmospheric Administration Physical Sciences Laboratory (NOAA Physical Sciences Laboratory, 2020), respectively.

### 2.4 Structural Equation Modeling for Under‐Ice Conditions

We constructed a structural equation model (SEM) that linked ice variables (ice‐on and ice‐off dates) with fall and spring mixing period lengths and underwater parameters (Grace et al., 2010). SEMs are a tool for examining causal relationships among multiple variables, enabling researchers to assess direct and indirect effects within ecological systems and test hypothesized pathways of influence (Grace et al., 2010). For each annual under‐ice period, we calculated the mean temperature in the shallow (1–3 m) and deep water zones (10–12 m). To estimate the magnitude of inverse stratification under ice, we calculated the difference in water density between 1 and 11 m (Δ density). We also calculated the mean heat content by calculating daily heat content in the lake using the temperature profile (Wetzel et al., 2000) and took the average across the under‐ice period for each year. We also calculated the length of the fall mixed period as the number of days between lake turnover and ice‐on and the spring mixed period as the number of days between ice‐off and the onset of summer stratification (Oleksy & Richardson, 2021). To account for varying magnitudes of ranges, we mean‐centered and unit‐variance scaled all variables. We built model relationships in a temporally linear fashion from fall to winter to spring to include regressions with causal relationships and covariances between variables that we expected to be collinear rather than causal. To visualize relationships from the optimal SEM, we calculated partial residual plots by calculating the residuals for each variable using the multiple regression equation from the SEM. We also plotted the component and component plus residual to show where the fitted regression line was located (Wood, 1973). All SEM statistics were calculated using the *lavaan* package (Rosseel, 2012), and visualizations were created using the *semPlot* (Epskamp, 2022), *ggplotify* (Yu, 2021), *ggnetwork* (Briatte, 2021), and *cowplot* (Wilke, 2020) packages.

### 2.5 High Frequency Analysis

For 2 winters (2016–2017 and 2017–2018), we matched water temperature with ice phenology and percent ice cover, extending out our time frame to precede ice formation and follow full ice clearance from the lake by several weeks. We used high‐frequency data to calculate additional metrics of ice‐on and ice‐off dates from Pierson et al. (2011). “Pierson ice‐on” was calculated as the first time that the upper sensor (0 m) records a temperature at least 0.1°C below the lowest (9 m) sensor temperature, and “Pierson ice‐off” was the first recorded temperature where the upper sensor was less than 0.1°C of the lowest sensor at the end of the winter season.

Given data availability, we focused on the formation of ice‐on for both winters. We used temperature data to calculate two metrics of inverse stratification: (a) temperature difference from 0 to 9 m and (b) Schmidt stability (Idso, 1973) using the *rLakeAnalyzer* R package (Read et al., 2011). For both metrics in the month of December for each winter, we calculated segmented regressions with 1 up to 7 breakpoints using the *segmented* R package (Muggeo, 2016). We selected the optimal fit indicated as the lowest residual standard error and highest *R* ^2^ values and selected the breakpoint before the steepest line as an indicator of statistically identifiable ice‐on date and time to the nearest 15 min. We compared that specific date and time to the 24‐hr period visually identified as the ice‐on day (first day with 100% ice cover). We compared the ice‐on data identified by both high‐frequency and visual ice‐on day to meteorological data including air temperature, wind speed, and barometric pressure from the local meteorological station.

## 3 Results

**Figure 1:**
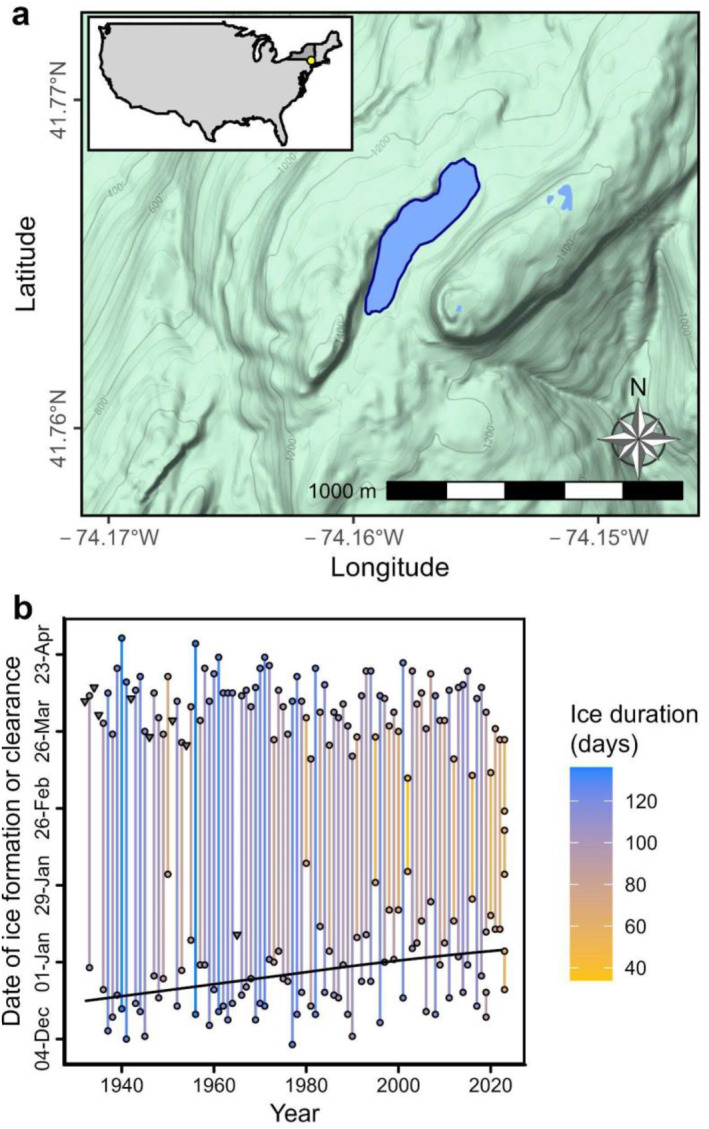
(a) Map of Mohonk Lake on the Northern Shawangunk Ridge. Inset indicates study site locations located in New York state, USA. Topography from Google Maps. (b) Ice phenology in Mohonk Lake from 1932 to 2023. Dates of ice‐on and ice‐off are plotted as points. A line segment connects the ice‐on and ice‐off in the same water year and the length and color gradient and point fill correspond to ice‐cover duration. The black line indicates significant Theil‐Sen's slope (*p* < 0.05). Triangles indicate years where only ice‐on or ice‐off was recorded.

**Figure 2:**
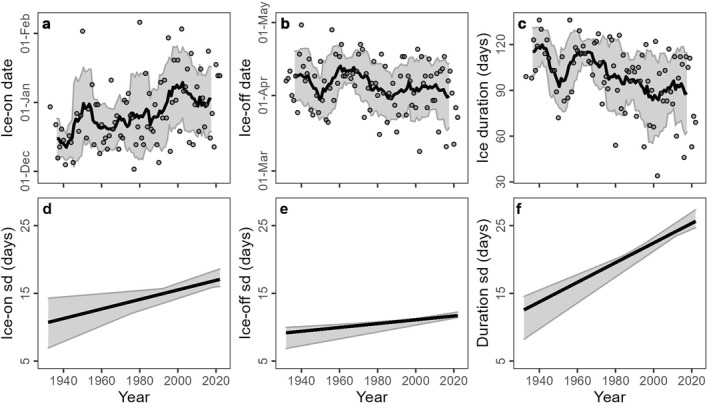
Ice phenology in Mohonk Lake from 1932 to 2022 for (a) ice‐on, (b) ice‐off, and (c) ice duration. For each figure, the 9‐year simple moving average is presented as the dark line, and the Bollinger Band (±10‐year rolling standard deviation) is the shaded area presented at the median year for each 10‐year window. Standard deviations for mean trends (slope and intercept) across all sequential windows (4–30 years)for (d) ice‐on, (e) ice‐off, and (f) ice duration with shaded areas representing minimum to 95% possible trend line fits for each year.

Between 1932 and 2023, ice‐on dates ranged between December 5th and February 8th with a median of December 27th. Ice‐off dates ranged between March 11 and May 2 with a median of April 9th. We observed that ice‐on is trending later (slope = 2.2 days decade^−1^, *p* = 0.001, Figure 1) but we did not detect a statistically significant trend toward earlier ice‐off over the 92‐year period of record (slope = −0.7 days decade^−1^, *p* = 0.13, Figure 1). Consequently, ice cover duration is getting shorter (slope = −3.6 days decade^−1^, *p* < 0.001, Figure 1). Across all the possible segment sizes (*n* = 4–30 years, Figure S4 in Supporting Information S1), ice‐on standard deviation increased by 0.7 days decade^−1^ (*p* < 0.001, minimum slope = 0.23 days decade^−1^, maximum slope = 1.2 days decade^−1^, Figure 2d). Ice‐off standard deviation increased by 0.28 days decade^−1^ (*p* < 0.001, minimum slope = 0.16 days decade^−1^, maximum slope = 0.5 days decade^−1^, Figure 2e). Ice duration variability increased by 1.45 days decade^−1^ (*p* < 0.001, minimum slope = 1.15 days decade^−1^, maximum slope = 1.96 days decade^−1^, Figure 2f).

**Figure 3:**
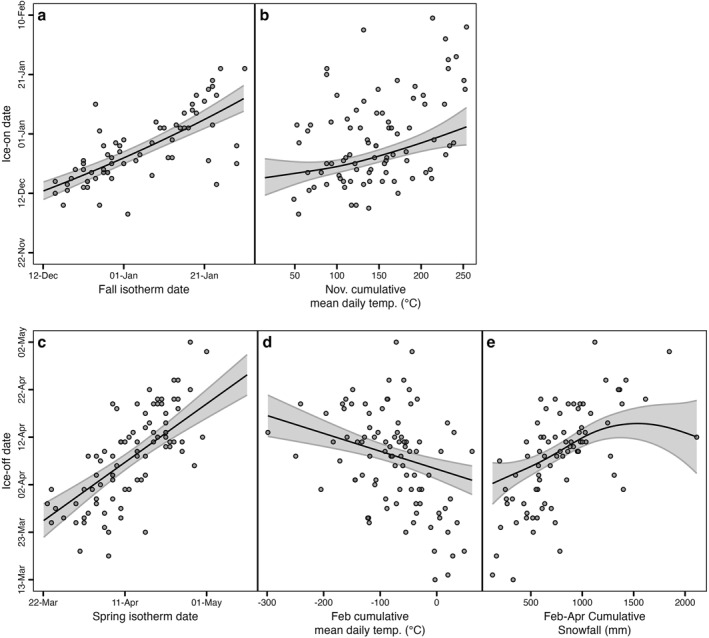
General additive model results for ice‐on date and ice‐off date. Ice‐on date was best explained by (a) 17 days after that the maximum daily air temperature fell below the 0°C air temperature isotherm and (b) cumulative mean daily air temperature in November (deviance explained 67.5%). Ice‐off date was best explained by (c) 29 days after the average daily air temperature exceeded the 4°C isotherm in the spring, (d) cumulative mean daily temperatures in February, and (e) cumulative snowfall between February and April (deviance explained 81.3%). For all panels, the points represent raw data, and the fitted curves are the predictions holding all other covariates at their median value.

Late fall and early winter temperatures were strong controls on the timing of ice‐on in Mohonk Lake (Figure 3). The best model explained 67.5% of the deviance in the ice‐on date and included a positive linear relationship with the timing of the fall 0°C isotherm date (Figure 3a, Table S2 in Supporting Information S1). A smaller proportion of the variance in ice‐on day of year (DOY) was attributed to cumulative daily mean air temperature in November (Figure 3b). The fall 0°C isotherm date shifted 2.1 days decade^−1^ later since the beginning of the ice monitoring record (*p* = 0.02; Table S3 in Supporting Information S1) but there was no trend in cumulative mean daily November air temperature (*p* > 0.05; Figure S6 in Supporting Information S1).

Ice‐off was controlled by a combination of late winter and spring precipitation and air temperature variables. The best model explained 81.3% of the variation in the ice‐off date (Table S2 in Supporting Information S1). The spring 4°C isotherm date had a largely positive linear effect on ice‐off DOY (Figure 3c). Cumulative mean daily air temperature in February also had a substantial effect on ice‐on DOY with warmer years resulting in earlier ice‐off (Figure 3d). The amount of snowfall between February and April (ranging between 127 and 2,113 mm) had a positive nonlinear impact on ice‐off DOY, such that years with greater amounts of snow were associated with later ice‐off, but above a certain snowfall threshold (∼1,250 mm), ice‐off DOY is unaffected (Figure 3e). The spring 4°C isotherm date shifted earlier by 1.4 days decade^−1^ (*p* = 0.001, *τ* = −3.24), and cumulative mean daily air temperature in February increased by 5.9°C decade^−1^ (*p* = 0.02, *τ* = 2.21), but cumulative snowfall between February and April did not change substantially (Table S3 in Supporting Information S1).

**Figure 4:**
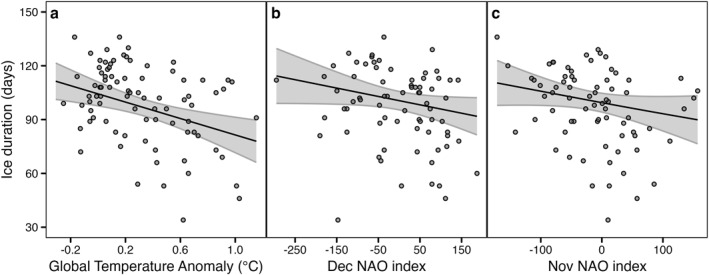
General Additive Model results for global drivers of ice duration. Ice duration was best explained by (a) global temperature anomaly, (b) December NAO index, and (c) November NAO index (deviance explained 24%). For all panels, the points represent raw data, and the fitted curves are the predictions holding all other covariates at their median value.

Variation in ice duration was partially explained by trends in global temperature anomaly and variability in November and December NAO cycles (Figure 4, deviance explained 24%). Higher global temperature anomalies since the early 20th century were associated with shorter ice duration. Years with negative November and December NAO indices were also correlated with shorter ice duration.

**Figure 5:**
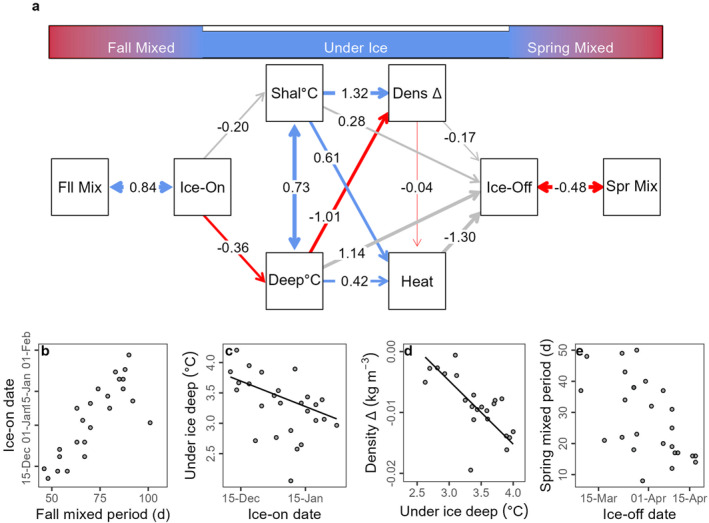
(a) A network plot is showing structural equation model results linking Mohonk Lake's fall‐mixed, under‐ice, and spring‐mixed seasons via ice phenology (Ice‐On and Ice‐Off dates). Starting with the length of fall mixing (Fll Mix) to under‐ice shallow (1–3 m: Shal°C) and deep (10–12 m: Deep°C) water temperatures, the water density difference between 1 and 11 m deep (Dens Δ), and mean under‐ice heat content (Heat) through the length of the spring mixing period (Spr Mix). Path coefficients are labeled on all edges with red lines indicating a significant negative relationship, blue lines indicating a significant positive relationship, gray lines indicating nonsignificant relationships, and the thickness of the edge arrow representing the strength of the relationship between mean‐centered and unit‐variance scaled variables. Single‐ended arrows indicate a causal regression while double‐ended arrows indicate covariance. From the structural equation model, covariance or partial residual plots for back‐transformed variables are displayed for four selected significant relationships including (b) the length of the fall mixing period and ice‐on date, (c) ice‐on date and deep under ice water temperatures, (d) deep under ice water temperatures and water density difference between 1 and 11 m deep and shallow under ice water temperatures, and (e) the length of the spring mixing period and ice‐off date. For causal regression relationships, component and component plus residual were plotted as a black line to show where the fitted regression line falls. With significant covariance relationships, no regression lines were plotted.

The selected SEM converged (Figure 5a) with a comparative fit index indicating improvement over the null model (Table S4 in Supporting Information S1). Longer fall mixing was correlated with later ice‐on dates (coefficient = 0.84, *p* = 0.001, Figure 5b). Later ice‐on dates resulted in lower deep water temperatures (coefficient = −0.36, *p* = 0.038, Figure 5c) but were not related to shallow average temperatures under ice (coefficient = −0.20, *p* = 0.29). Warmer deep temperatures resulted in more negative water density differences (coefficient = −1.01, *p* = 0.003, Figure 5d) whereas warmer shallow temperatures resulted in small water density differences (coefficient = 1.32, *p* < 0.001). Warmer shallow (coefficient = 0.61, *p* < 0.001) and deep (coefficient = 0.42, *p* < 0.001) temperatures resulted in higher mean heat content. Ice‐off date was negatively correlated with the length of the spring mixing period (coefficient = −0.48, *p* = 0.014, Figure 5e).

**Figure 6:**
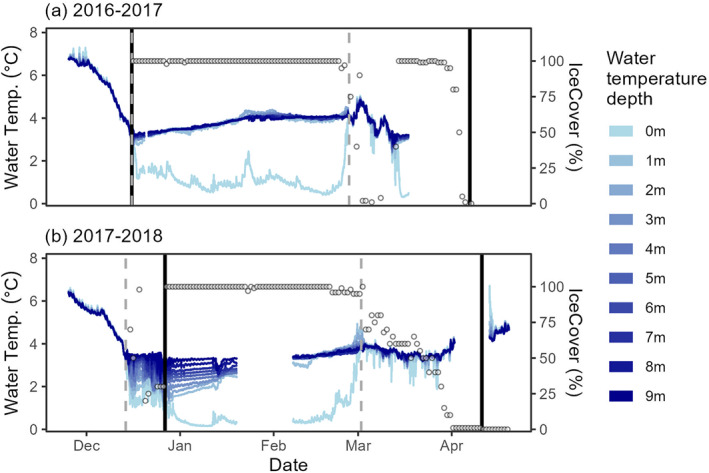
High‐frequency (every 15 min) data from (a) 2016–2017 and (b) 2017–2018 winters. The blue line shade indicates the depth of each temperature sensor (left *y*‐axis). Open circles indicate the daily percentage of ice cover (right *y*‐axis). The vertical solid line indicates visually assessed ice‐on and ice‐off whereas the vertical dotted line indicates the ice‐on and ice‐off based on temperature differences between the top and bottom temperature sensors (Pierson et al., 2011).

**Figure 7:**
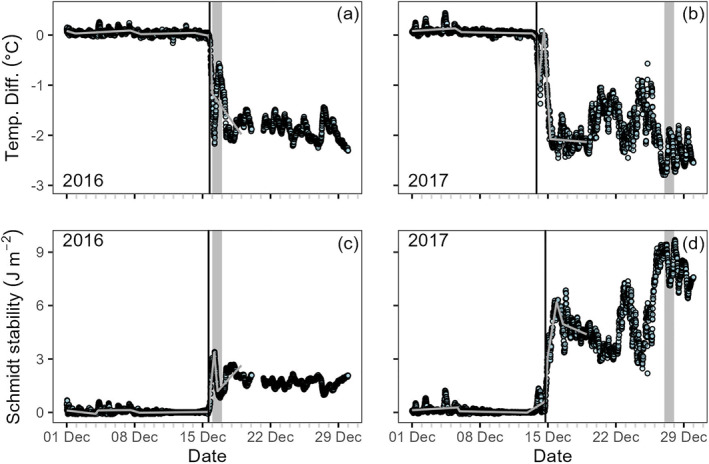
High frequency (15 min) metrics of inverse stratification including temperature difference between the upper (0 m) and lowest (9 m) temperature sensors in December (a) 2016 and (b) 2017 and the Schmidt stability in December (c) 2016 and (d) 2017. The vertical black bar indicates the steepest line of change for all metrics as an indicator of statistically identifiable ice‐on date and time to the nearest 15 min. The gray bar indicates the day (full 24‐hr period) identified from visual assessment as the first day of the winter with 100% ice cover. The gray lines are the best fit segmented regressions.

Visual ice‐on matched the Pierson ice‐on identified by high‐frequency data exactly for winter 2016–2017 (16 December 2016, Figure 6a). However, in winter 2017–2018, visual ice‐on (27 December 2017) was 2 weeks after the Pierson ice‐on (14 December 2017, Figure 6b). Visual ice‐off for both years was more than a month later than ice‐off identified using high‐frequency data (Figure 6). We can identify the exact hour that inverse stratification/ice forms using the high frequency data (Figure 7). The two metrics of temperature difference and Schmidt stability were consistent with each other and identified night times as when ice‐on occurred. In winter 2016, temp difference indicated ice‐on occurring at 15 December 2016 22:30; stability indicated ice‐on occurring at 15 December 2016 19:45:00 less than 3 hr apart. Both were consistent with visual ice‐on (Figures 7a and 7c). In winter 2017, temp difference indicated ice‐on occurring at 14 December 2017 00:45; stability indicated ice‐on occurring at 14 December 2017 22:45 still within a day of each other. However, these were about 2 weeks earlier than visual ice‐on (Figures 7b and 7d). For both years, ice‐on required well‐below freezing and consistently dropping air temperatures (Figures S7a and S7b in Supporting Information S1). There was less evidence of the contribution of wind (Figures S7c and S7d in Supporting Information S1), but dropping or low barometric pressure could facilitate or initiate lake freezing (Figures S7e and S7f in Supporting Information S1). In March of the year with intermittent ice cover (2016–2017), the lake vented out accumulated heat during the period of ice loss and later reformed at a lower overall heat content (Figure 6).

## 4 Discussion

Decreases in lake ice cover duration and increases in interannual variability in ice duration of seasonally ice‐covered lakes worldwide (Richardson et al., 2024; Sharma et al., 2021) highlight a need to understand the implications for lake thermal characteristics both during the shrinking under‐ice period and the potential for carryover effects into the subsequent spring and summer (Dugan, 2021; Lewis & Carey, 2024). In this study, we investigated the changes to ice phenology and attributed climatic drivers to the variability in a 92‐year record of lake ice cover. We leveraged weekly temperature profiles that spanned the last third of that record and 2 years of high‐frequency temperate profiles during the under‐ice period to understand the implications of changing lake ice phenology on the thermal characteristics of this lake. Below, we place our results in a broader context of Northern Hemisphere lake ice loss, illustrate the potential pitfalls of relying on binary ice on or off metrics, and discuss the potential implications for lake ecosystem function under a warmer and increasingly variable future.

### 4.1 Global and Local Drivers of Ice Change

Mohonk Lake experienced decreases in winter ice‐cover that amounted to a loss of approximately 1 month of winter ice cover over a 92‐year period. Trends in ice duration in Mohonk Lake were driven largely by later ice formation and not earlier clearance; although ice‐off was not trending earlier, interannual variability in ice‐cover increased as in many other seasonally ice‐covered lakes (Benson et al., 2012; Richardson et al., 2024). Overall, decreased ice duration was related to the interplay between global climate change and teleconnections, which influenced local weather drivers, and, in turn, ice phenology. Specifically, shorter ice duration was associated with higher global temperature anomalies, which were captured in variables such as increasing fall, winter, and spring air temperatures (Table S3 in Supporting Information S1). Additional variation in ice duration was explained by the North Atlantic Oscillation (NAO) in November and December. In winters with positive NAO, the northeastern U.S. experiences mild winters whereas negative NAO is associated with cold‐air outbreaks and strong storms, which may promote earlier ice formation or thicker ice cover, respectively. Teleconnections like El Niño‐Southern Oscillation and NAO have been similarly shown to modify both ice phenology and summer stratification in lakes (Bai et al., 2012; Oleksy & Richardson, 2021; Sharma & Magnuson, 2014). For example, winter NAO has a substantial effect on spring hypolimnetic temperatures that ultimately carry into the stratified period in deep European lakes (Dokulil et al., 2006).

In Mohonk Lake, ice‐on and ice duration changed twice as fast compared to means from other northern hemisphere lakes (1.2 days decade^−1^ later and 1.7 days decade^−1^ shorter, respectively; Sharma et al., 2021), and the high rate of change was similar to lakes in the southern Rocky Mountains and the European Alps, which have some of the most rapid lake ice losses globally (6.6 and 3.6 days decade^−1^, respectively; Christianson et al., 2021; Kainz et al., 2017). The rapid changes in ice phenology were tightly linked to regional and local climatic shifts. The date when air temperature crossed thresholds that precipitated ice formation (e.g., 0°C) was getting later each year (2 days decade^−1^; Table S3 in Supporting Information S1). Consecutive cold days, in combination with cumulative mean daily temperature in November, intuitively explained about two‐thirds of the variability in ice formation timing on Mohonk Lake and have resulted in substantially later ice formation over time. Wind speed is another critical factor explaining lake ice formation (Bartosiewicz et al., 2021); wind can modulate lake heat transfer (Read et al., 2012) and disrupt ice formation when it is still thin (Kirillin et al., 2012) but is missing from our model due to a lack of data availability across the majority of the record.

Although ice‐off timing in the spring has not trended earlier, contrary to our expectations, it has been growing increasingly more variable in recent decades; preceding seasonal weather patterns explained the majority of that interannual variability. Ice‐off in Mohonk was controlled by a combination of late winter and early spring air temperatures and cumulative snowfall consistent with mountain lakes in the western United States (Caldwell et al., 2021; Preston et al., 2016; Smits et al., 2021). Warmer conditions in late winter and spring and increases in solar radiation can accelerate ice breakup (Sharma et al., 2013), but this relationship is likely modified by the amount of precipitation that falls on the lake particularly as snow. In years with more late winter and spring snowfall, lake ice likely persists longer because the lake has higher albedo with the accumulation of snow and associated cold temperatures (Cavaliere et al., 2021; Kouraev et al., 2007; Tronstad et al., 2024).

### 4.2 Under‐Ice Water Temperatures

Since Mohonk is a bedrock‐constrained lake in a small watershed with no surface or groundwater inflows, under‐ice thermal dynamics are likely regulated by a combination of ice duration and ice clarity, which determine the degree of radiation‐driven convection and mixing (Bruesewitz et al., 2015; Cavaliere et al., 2021). Later ice formation was associated with lower deep average water temperatures and thus resulted in cooler well‐mixed water columns that did not inversely stratify (Woolway, Denfeld, et al., 2022; Woolway, Sharma, & Smol, 2022). In Mohonk Lake, in winters with shorter ice duration (driven by later ice‐on), under‐ice inverse stratification was weaker and shorter. Both weaker inverse stratification and shorter ice duration will shorten the phase of stable inverse stratification under the ice (Bruesewitz et al., 2015; Kirillin et al., 2012). The phase before ice‐off occurs in the spring can both last weeks or longer with deepening convective layers driven by clear ice and cycles of daily solar radiation (Bruesewitz et al., 2015; Kirillin et al., 2012; Yang et al., 2017). Snowmelt, while important in mountain lakes (Smits et al., 2020) or groundwater intrusion lakes (Kirillin et al., 2012), likely plays a minimal role in regulating under‐ice mixing in Mohonk.

Although none of the under‐ice thermal metrics were causally linked to ice‐off date, our results point to the potential for ice phenology to impact spring thermal dynamics (Dugan, 2021; Li et al., 2022). Specifically, in years with longer ice seasons, the lake quickly stratified following ice clearance; although the spring mixed period was shorter, the onset of stratification was indeed later than average (Oleksy & Richardson, 2021). In contrast, when ice clearance occurred earlier, the spring mixing period was longer, but the onset of stratification was earlier than average with warmer hypolimnetic temperatures during the spring mixed‐period as well as during the stratified summer months (Oleksy & Richardson, 2021). Spring ice conditions are important antecedent conditions that set the stage for spring mixed period and summer thermal dynamics, a concept increasingly referred to as ecological memory (e.g., Dugan, 2021; Lewis & Carey, 2024).

The two winters with additional high‐frequency temperature profiles provided some additional insights into the relationship between ice phenology observations and under‐ice thermal characteristics. Although historical visual lake ice records are an invaluable indicator of lake responses to climate change (Magnuson et al., 2000), there is considerable variability in how observers define “ice on” or “ice off” (Sharma et al., 2022), which may not always align with the true thermal properties of the lake. For instance, at Mohonk Lake, we define “ice on” as the first day with 100% ice cover and “ice off” as the first day in the spring with 0% ice cover while other lake ice monitoring programs use a variety of different metrics, for example, ice‐off occurs when a lake is navigable by boat even with some ice coverage (Sharma et al., 2022). One lesson learned by this binary phenology observation is that visual observations of ice might not always match up to the under‐ice thermodynamics. In 2016 where ice formation occurred quickly, the day we recorded 100% ice coverage (“ice on”) coincided with two metrics of inverse stratification, although in 2017, the lake was inversely stratified nearly 2 weeks before the lake reached 100% ice cover (Figure 6). Similarly, inverse stratification ceased and the lake thermally mixed days (spring 2017) or weeks (spring 2018) prior to the loss of ice complete ice cover in the lake (Figure 6). Questions remain as to how intermittent ice cover, the speed of ice loss and ice quality drive (e.g., clear ice, white ice, or snow on ice) interact to affect under‐ice thermal dynamics and other temperature and mixing‐dependent biogeochemical processes in lakes.

### 4.3 Ecological Implications

Through changes in thermal dynamics, the loss of lake ice has the potential to alter many different biogeochemical and ecological dynamics in lakes. With later ice formation in the fall, more phytoplankton can grow and increase zooplankton overwintering success, dampening the spring phytoplankton bloom (Hébert et al., 2021). In spring, antecedent winter conditions alter the successional dynamics of phytoplankton assemblages and in turn can result in mismatched phenology of herbivorous zooplankton (Hrycik et al., 2022). With longer mixed seasons and earlier onset of stratification, Mohonk Lake would likely have exacerbated mismatches of zooplankton and phytoplankton spring phenology. Additionally, shorter duration of ice cover and thinner ice promotes higher under‐ice metabolism, which can account for a substantial amount of annual net ecosystem production (e.g., Brentrup et al., 2021).

Mohonk Lake appears to be at risk of transitioning from being classified as dimictic to monomictic in the near future. For example, the 2022–2023 winter had three periods of ice freeze and melt (Figure 1), the first time this had happened in this nearly century‐long ice record. There is potential for future winters with shortening ice phases, no ice, and varying degrees of ice quality (Sharma et al., 2019, 2021; Weyhenmeyer et al., 2022). Winter limnological studies like this one are critical in “closing the loop” between under‐ice and ice‐free seasons (Salonen et al., 2009) and underscore the repercussions of changing winter ice phenology on lake ecosystem dynamics.

## Supporting information

Supporting Information S1

## Data Availability

All data and scripts needed to recreate the analysis are publicly available and hosted on Zenodo (https://doi.org/10.5281/zenodo.14037563). Ice phenology records are published by Sharma et al. (2022). Under‐ice temperature profile data are published by Mohonk et al. (2020) (https://doi.org/10.6073/pasta/7b67399344129afc63cd57e99e778160). Daily air temperature and precipitation data from the Mohonk Preserve Weather station are available from the US Weather Bureau/National Weather Service rain gauge (Network ID GHCND:USC00305426. https://www.ncei.noaa.gov/access/past‐weather/41.77294529685411,‐74.16625081576478,41.76294158679213,‐74.14166120041256).

## References

[jgrg22872-bib-0001] Adrian, R. , Walz, N. , Hintze, T. , Hoeg, S. , & Rusche, R. (1999). Effects of ice duration on plankton succession during spring in a shallow polymictic lake. Freshwater Biology, 41(3), 621–634. 10.1046/j.1365-2427.1999.00411.x

[jgrg22872-bib-0002] Bai, X. , Wang, J. , Sellinger, C. , Clites, A. , & Assel, R. (2012). Interannual variability of Great Lakes ice cover and its relationship to NAO and ENSO. Journal of Geophysical Research, 117(C3), C03002. 10.1029/2010JC006932

[jgrg22872-bib-0003] Bartosiewicz, M. , Ptak, M. , Woolway, R. I. , & Sojka, M. (2021). On thinning ice: Effects of atmospheric warming, changes in wind speed and rainfall on ice conditions in temperate lakes (Northern Poland). Journal of Hydrology, 597, 125724. 10.1016/j.jhydrol.2020.125724

[jgrg22872-bib-0004] Benson, B. J. , Magnuson, J. J. , Jensen, O. P. , Card, V. M. , Hodgkins, G. , Korhonen, J. , et al. (2012). Extreme events, trends, and variability in Northern Hemisphere lake‐ice phenology (1855–2005). Climatic Change, 112(2), 299–323. 10.1007/s10584-011-0212-8

[jgrg22872-bib-0005] Beyene, M. T. , & Jain, S. (2015). Wintertime weather‐climate variability and its links to early spring ice‐out in Maine lakes. Limnology & Oceanography, 60(6), 1890–1905. 10.1002/lno.10148

[jgrg22872-bib-0006] Bollinger, J. (1992). Using bollinger bands. Stocks & Commodities, 10(2), 47–51.

[jgrg22872-bib-0007] Brentrup, J. A. , Richardson, D. C. , Carey, C. C. , Ward, N. K. , Bruesewitz, D. A. , & Weathers, K. C. (2021). Under‐ice respiration rates shift the annual carbon cycle in the mixed layer of an oligotrophic lake from autotrophy to heterotrophy. Inland Waters, 11(1), 114–123. 10.1080/20442041.2020.1805261

[jgrg22872-bib-0008] Briatte, F. (2021). ggnetwork: Geometries to plot networks with “ggplot2”. Retrieved from https://CRAN.R‐project.org/package=ggnetwork

[jgrg22872-bib-0009] Bronaugh, D. , & Consortium, A. W. for the P. C. I . (2019). zyp: Zhang + Yue‐Pilon trends package (0.10‐1.1). Retrieved from https://CRAN.R‐project.org/package=zyp

[jgrg22872-bib-0010] Brotzge, J. A. , Wang, J. , Thorncroft, C. D. , Joseph, E. , Bain, N. F. , Freedman, J. M. , et al. (2020). A technical overview of the New York State Mesonet standard network. Journal of Atmospheric and Oceanic Technology, 37(10), 1827–1845. 10.1175/jtech-d-19-0220.1

[jgrg22872-bib-0011] Bruesewitz, D. A. , Carey, C. C. , Richardson, D. C. , & Weathers, K. C. (2015). Under‐ice thermal stratification dynamics of a large, deep lake revealed by high‐frequency data. Limnology & Oceanography, 60(2), 347–359. 10.1002/lno.10014

[jgrg22872-bib-0012] Caldwell, T. J. , Chandra, S. , Albright, T. P. , Harpold, A. A. , Dilts, T. E. , Greenberg, J. A. , et al. (2021). Drivers and projections of ice phenology in mountain lakes in the western United States. Limnology & Oceanography, 66(3), 995–1008. 10.1002/lno.11656

[jgrg22872-bib-0013] Cavaliere, E. , Fournier, I. B. , Hazuková, V. , Rue, G. P. , Sadro, S. , Berger, S. A. , et al. (2021). The lake ice continuum concept: Influence of winter conditions on energy and ecosystem dynamics. Journal of Geophysical Research: Biogeosciences, 126(11), 1–20. 10.1029/2020JG006165 37089664

[jgrg22872-bib-0014] Christianson, K. R. , Loria, K. A. , Blanken, P. D. , Caine, N. , & Johnson, P. T. J. (2021). On thin ice: Linking elevation and long‐term losses of lake ice cover. Limnology and Oceanography Letters, 6(2), 77–84. 10.1002/lol2.10181

[jgrg22872-bib-0015] Dokulil, M. T. , Jagsch, A. , George, G. D. , Anneville, O. , Jankowski, T. , Wahl, B. , et al. (2006). Twenty years of spatially coherent deepwater warming in lakes across Europe related to the North Atlantic Oscillation. Limnology & Oceanography, 51(6), 2787–2793. 10.4319/lo.2006.51.6.2787

[jgrg22872-bib-0016] Dugan, H. A. (2021). A comparison of ecological memory of lake ice‐off in eight north‐temperate lakes. Journal of Geophysical Research: Biogeosciences, 126(6), 1–13. 10.1029/2020jg006232 37089664

[jgrg22872-bib-0017] Epskamp, S. (2022). semPlot: Path diagrams and visual analysis of various SEM packages’ output. Retrieved from https://CRAN.R‐project.org/package=semPlot

[jgrg22872-bib-0018] Grace, J. B. , Anderson, T. M. , Olff, H. , & Scheiner, S. M. (2010). On the specification of structural equation models for ecological systems. Ecological Monographs, 80(1), 67–87. 10.1890/09-0464.1

[jgrg22872-bib-0019] Hastie, T. , & Tibshirani, R. (1987). Generalized additive models, cubic splines and personalized likelihood. University of Toronto, Department of Statistics.

[jgrg22872-bib-0020] Hébert, M.‐P. , Beisner, B. E. , Rautio, M. , & Fussmann, G. F. (2021). Warming winters in lakes: Later ice onset promotes consumer overwintering and shapes springtime planktonic food webs. Proceedings of the National Academy of Sciences of the United States of America, 118(48). 10.1073/PNAS.2114840118 PMC8694056 34810251

[jgrg22872-bib-0021] Higgins, S. N. , Desjardins, C. M. , Drouin, H. , Hrenchuk, L. E. , & van der Sanden, J. J. (2021). The role of climate and lake size in regulating the ice phenology of boreal lakes. Journal of Geophysical Research: Biogeosciences, 126(3), 1–11. 10.1029/2020JG005898 37089664

[jgrg22872-bib-0022] Hrycik, A. R. , McFarland, S. , Morales‐Williams, A. , & Stockwell, J. D. (2022). Winter severity shapes spring plankton succession in a small, eutrophic lake. Hydrobiologia, 849(9), 2127–2144. 10.1007/s10750-022-04854-4

[jgrg22872-bib-0023] Idso, S. B. (1973). On the concept of lake stability. Limnology & Oceanography, 18(4), 681–683. 10.4319/lo.1973.18.4.0681

[jgrg22872-bib-0024] Jansen, J. , MacIntyre, S. , Barrett, D. C. , Chin, Y.‐P. , Cortés, A. , Forrest, A. L. , et al. (2021). Winter limnology: How do hydrodynamics and biogeochemistry shape ecosystems under ice? Journal of Geophysical Research: Biogeosciences, 126(6), e2020JG006237. 10.1029/2020JG006237

[jgrg22872-bib-0025] Kainz, M. J. , Ptacnik, R. , Rasconi, S. , & Hager, H. H. (2017). Irregular changes in lake surface water temperature and ice cover in subalpine Lake Lunz, Austria. Inland Waters, 7(1), 27–33. 10.1080/20442041.2017.1294332

[jgrg22872-bib-0026] Kirillin, G. , Leppäranta, M. , Terzhevik, A. , Granin, N. , Bernhardt, J. , Engelhardt, C. , et al. (2012). Physics of seasonally ice‐covered lakes: A review. Aquatic Sciences, 74(4), 659–682. 10.1007/s00027-012-0279-y

[jgrg22872-bib-0027] Kouraev, A. V. , Semovski, S. V. , Shimaraev, M. N. , Mognard, N. M. , Legrésy, B. , & Rémy, F. (2007). The ice regime of Lake Baikal from historical and satellite data: Relationship to air temperature, dynamical, and other factors. Limnology & Oceanography, 52(3), 1268–1286. 10.4319/lo.2007.52.3.1268

[jgrg22872-bib-0028] Lewis, A. S. L. , & Carey, C. C. (2024). Ecological memory of spring air temperature drives summer water quality dynamics in temperate lakes. Authorea. 10.22541/au.171812883.31377074/v1

[jgrg22872-bib-0029] Li, X. , Peng, S. , Xi, Y. , Woolway, R. I. , & Liu, G. (2022). Earlier ice loss accelerates lake warming in the Northern Hemisphere. Nature Communications, 13(1), 5156. Article 1. 10.1038/s41467-022-32830-y PMC9440048 36056046

[jgrg22872-bib-0030] Magnuson, J. J. , Wynne, R. H. , Benson, B. J. , & Robertson, D. M. (2000). Lake and river ice as a powerful indicator of past and present climates. SIL Proceedings, 1922‐2010, 27(5), 2749–2756. 10.1080/03680770.1998.11898166

[jgrg22872-bib-0031] Mohonk, P. , Belardo, C. , Feldsine, N. , Huth, P. , Long, E. C. , Napoli, M. , et al. (2020). Weekly and high frequency temperature profile data and Secchi depth, Mohonk Lake, NY, USA, 1985 to 2017 version 1. Environmental Data Initiative. 10.6073/pasta/7b67399344129afc63cd57e99e778160

[jgrg22872-bib-0032] Muggeo, V. M. R. (2016). Testing with a nuisance parameter present only under the alternative: A score‐based approach with application to segmented modeling. Journal of Statistical Computation and Simulation, 86(15), 3059–3067. 10.1080/00949655.2016.1149855

[jgrg22872-bib-0033] National Weather Service . (2020). Climate prediction center—Teleconnections: North Atlantic Oscillation. Retrieved from https://www.cpc.ncep.noaa.gov/products/precip/CWlink/pna/nao.shtml

[jgrg22872-bib-0034] NOAA . (2020). Global surface temperature anomalies | monitoring references. National Centers for Environmental Information (NCEI). Retrieved from https://www.ncdc.noaa.gov/monitoring‐references/faq/anomalies.php#anomalies

[jgrg22872-bib-0035] NOAA Physical Sciences Laboratory . (2020). Climate indices: Monthly atmospheric and ocean time series. Retrieved from https://psl.noaa.gov/data/climateindices/

[jgrg22872-bib-0036] Oleksy, I. A. , & Richardson, D. C. (2021). Climate change and teleconnections amplify lake stratification with differential local controls of surface water warming and deep water cooling. Geophysical Research Letters, 48(5), 1–11. 10.1029/2020GL090959

[jgrg22872-bib-0037] Pedersen, T. L. (2022). patchwork: The composer of plots. Retrieved from https://CRAN.R‐project.org/package=patchwork

[jgrg22872-bib-0038] Pierson, D. C. , Weyhenmeyer, G. A. , Arvola, L. , Benson, B. , Blenckner, T. , Kratz, T. , et al. (2011). An automated method to monitor lake ice phenology. Limnology and Oceanography: Methods, 9(2), 74–83. 10.4319/lom.2010.9.0074

[jgrg22872-bib-0039] Pohlert, T. (2020). trend: Non‐parametric trend tests and change‐point detection (1.1.2). Retrieved from https://CRAN.R‐project.org/package=trend

[jgrg22872-bib-0040] Preston, D. L. , Caine, N. , McKnight, D. M. , Williams, M. W. , Hell, K. , Miller, M. P. , et al. (2016). Climate regulates alpine lake ice cover phenology and aquatic ecosystem structure. Geophysical Research Letters, 43(10), 5353–5360. 10.1002/2016GL069036

[jgrg22872-bib-0041] Prowse, T. , Alfredsen, K. , Beltaos, S. , Bonsal, B. R. , Bowden, W. B. , Duguay, C. R. , et al. (2011). Effects of changes in Arctic lake and river ice. Ambio, 40(1), 63–74. 10.1007/s13280-011-0217-6

[jgrg22872-bib-0042] R Core Team . (2022). R: A language and environment for statistical computing. R Foundation for Statistical Computing. Retrieved from https://www.R‐project.org/

[jgrg22872-bib-0043] Read, J. S. , Hamilton, D. P. , Desai, A. R. , Rose, K. C. , MacIntyre, S. , Lenters, J. D. , et al. (2012). Lake‐size dependency of wind shear and convection as controls on gas exchange. Geophysical Research Letters, 39(9), L09405. 10.1029/2012GL051886

[jgrg22872-bib-0044] Read, J. S. , Hamilton, D. P. , Jones, I. D. , Muraoka, K. , Winslow, L. A. , Kroiss, R. , et al. (2011). Derivation of lake mixing and stratification indices from high‐resolution lake buoy data. Environmental Modelling & Software, 26(11), 1325–1336. 10.1016/j.envsoft.2011.05.006

[jgrg22872-bib-0045] Richardson, D. C. , Charifson, D. M. , Davis, B. A. , Farragher, M. J. , Krebs, B. S. , Long, E. C. , et al. (2018). Watershed management and underlying geology in three lakes control divergent responses to decreasing acid precipitation. Inland Waters, 8(1), 70–81. 10.1080/20442041.2018.1428428

[jgrg22872-bib-0046] Richardson, D. C. , Filazzola, A. , Woolway, R. I. , Imrit, M. A. , Bouffard, D. , Weyhenmeyer, G. A. , et al. (2024). Non‐linear responses in interannual variability of lake ice with climate change. Limnology & Oceanography, 69(4), 789–801. 10.1002/lno.12527

[jgrg22872-bib-0047] Robertson, D. M. , Ragotzkie, R. A. , & Magnuson, J. J. (1992). Lake ice records used to detect historical and future climatic changes. Climatic Change, 21(4), 407–427. 10.1007/BF00141379

[jgrg22872-bib-0048] Rosseel, Y. (2012). lavaan: An R package for structural equation modeling. Journal of Statistical Software, 48(2). 10.18637/jss.v048.i02

[jgrg22872-bib-0049] Salonen, K. , Leppäranta, M. , Viljanen, M. , & Gulati, R. D. (2009). Perspectives in winter limnology: Closing the annual cycle of freezing lakes. Aquatic Ecology, 43(3), 609–616. 10.1007/s10452-009-9278-z

[jgrg22872-bib-0050] Sharma, S. , Blagrave, K. , Magnuson, J. J. , O’Reilly, C. M. , Oliver, S. , Batt, R. D. , et al. (2019). Widespread loss of lake ice around the Northern Hemisphere in a warming world. Nature Climate Change, 9(3), 227–231. Article 3. 10.1038/s41558-018-0393-5

[jgrg22872-bib-0051] Sharma, S. , Filazzola, A. , Nguyen, T. , Imrit, M. A. , Blagrave, K. , Bouffard, D. , et al. (2022). Long‐term ice phenology records spanning up to 578 years for 78 lakes around the Northern Hemisphere. Scientific Data, 9(1), 318. Article 1. 10.1038/s41597-022-01391-6 35710905 PMC9203534

[jgrg22872-bib-0052] Sharma, S. , & Magnuson, J. J. (2014). Oscillatory dynamics do not mask linear trends in the timing of ice breakup for Northern Hemisphere lakes from 1855 to 2004. Climatic Change, 124(4), 835–847. 10.1007/s10584-014-1125-0

[jgrg22872-bib-0053] Sharma, S. , Magnuson, J. J. , Mendoza, G. , & Carpenter, S. R. (2013). Influences of local weather, large‐scale climatic drivers, and the ca. 11 year solar cycle on lake ice breakup dates; 1905–2004. Climatic Change, 118(3), 857–870. 10.1007/s10584-012-0670-7

[jgrg22872-bib-0054] Sharma, S. , Richardson, D. C. , Woolway, R. I. , Imrit, M. A. , Bouffard, D. , Blagrave, K. , et al. (2021). Loss of ice cover, shifting phenology, and more extreme events in Northern Hemisphere lakes. Journal of Geophysical Research: Biogeosciences, 126(10), e2021JG006348. 10.1029/2021JG006348

[jgrg22872-bib-0055] Smits, A. P. , Gomez, N. W. , Dozier, J. , & Sadro, S. (2021). Winter climate and lake morphology control ice phenology and under‐ice temperature and oxygen regimes in mountain lakes. Journal of Geophysical Research: Biogeosciences, 126(8), e2021JG006277. 10.1029/2021JG006277

[jgrg22872-bib-0056] Smits, A. P. , Macintyre, S. , & Sadro, S. (2020). Snowpack determines relative importance of climate factors driving summer lake warming. Limnology and Oceanography Letters, 5(3), 271–279. Article 3. 10.1002/lol2.10147

[jgrg22872-bib-0057] Tronstad, L. , Oleksy, I. , Pomeranz, J. P. , Preston, D. , Gianniny, G. , Cook, K. , et al. (2024). Despite a century of warming, increased snowfall has buffered the ice phenology of North America’s largest high‐elevation lake against climate change. Environmental Research Letters, 19(5), 054028. 10.1088/1748-9326/ad3bd1

[jgrg22872-bib-0058] Wetzel, R. G. , Likens, G. E. , Wetzel, R. G. , & Likens, G. E. (2000). The heat budget of lakes. Limnological Analyses, 45–56. 10.1007/978-1-4757-3250-4_4

[jgrg22872-bib-0059] Weyhenmeyer, G. A. , Obertegger, U. , Rudebeck, H. , Jakobsson, E. , Jansen, J. , Zdorovennova, G. , et al. (2022). Towards critical white ice conditions in lakes under global warming. Nature Communications, 13(1), 4974. Article 1. 10.1038/s41467-022-32633-1 PMC9411540 36008420

[jgrg22872-bib-0060] Wickham, H. (2016). ggplot2: Elegant graphics for data analysis. Springer‐Verlag. Retrieved from https://ggplot2.tidyverse.org

[jgrg22872-bib-0061] Wilke, C. O. (2020). cowplot: Streamlined plot theme and plot annotations for “ggplot2”. Retrieved from https://CRAN.R‐project.org/package=cowplot

[jgrg22872-bib-0062] Wilkinson, G. M. , Walter, J. , Fleck, R. , & Pace, M. L. (2020). Beyond the trends: The need to understand multiannual dynamics in aquatic ecosystems. Limnology and Oceanography Letters, 5(4), 281–286. lol2.10153. 10.1002/lol2.10153

[jgrg22872-bib-0063] Wood, F. S. (1973). The use of individual effects and residuals in fitting equations to data. Technometrics, 15(4), 677–695. 10.1080/00401706.1973.10489104

[jgrg22872-bib-0064] Wood, S. (2019). mgcv: Mixed GAM computation vehicle with automatic smoothness estimation (1.8‐31). Retrieved from https://CRAN.R‐project.org/package=mgcv

[jgrg22872-bib-0065] Wood, S. N. (2017). Generalized additive models (2nd ed.). Chapman and Hall/CRC.

[jgrg22872-bib-0066] Woolway, R. I. , Denfeld, B. , Tan, Z. , Jansen, J. , Weyhenmeyer, G. A. , & La Fuente, S. (2022). Winter inverse lake stratification under historic and future climate change. Limnology and Oceanography Letters, 7(4), 302–311. 10.1002/lol2.10231

[jgrg22872-bib-0067] Woolway, R. I. , Sharma, S. , & Smol, J. P. (2022). Lakes in hot water: The impacts of a changing climate on aquatic ecosystems. BioScience, 72(11), 1050–1061. 10.1093/biosci/biac052 36325103 PMC9618276

[jgrg22872-bib-0068] Yang, B. , Wells, M. G. , McMeans, B. C. , Dugan, H. A. , Rusak, J. A. , Weyhenmeyer, G. A. , et al. (2021). A new thermal categorization of ice‐covered lakes. Geophysical Research Letters, 48(3), e2020GL091374. 10.1029/2020GL091374

[jgrg22872-bib-0069] Yang, B. , Young, J. , Brown, L. , & Wells, M. (2017). High‐frequency observations of temperature and dissolved oxygen reveal under‐ice convection in a large lake. Geophysical Research Letters, 44(24), 12218–12226. 10.1002/2017GL075373

[jgrg22872-bib-0070] Yu, G. (2021). ggplotify: Convert plot to “grob” or “ggplot” object. Retrieved from https://CRAN.R‐project.org/package=ggplotify

