# Supplementary material for: Shorter Ice Duration and Changing Phenology Influence Under‐Ice Lake Temperature Dynamics
Source: J Geophys Res Biogeosci. 2024 Nov 23;129(11):e2024JG008382. doi: 10.1029/2024JG008382 (PMC11584988; doi:10.1029/2024JG008382)
Supplement: Supplementary file 1 — Supporting Information S1 [file JGRG-129-0-s001.pdf]

Supplemental materials

Title: Shorter ice duration and changing phenology influence under-ice lake temperature dynamics

Running head: Changing under-ice temperature dynamics

Author names: Isabella A. Oleksy*[a], David C. Richardson[b]

Sequential standard deviation windows: To assess changing variability of ice phenology, we evaluated 3 to 13-year windows of ice-on, ice-off, and ice duration for sequential windows as follows. First, we identified Y different series of standard deviations (Y years where Y=3,...,13) starting with 1932 through 1932+Y-1, beyond which the windows would repeat. Next, for each series, we calculated the standard deviation for sequential windows of Y years. For example, there are four unique versions of the 4-year windows (Fig. S1), nine unique versions of the 9-year windows (Fig. S2), and 13 unique versions of the 13-year windows (Fig. S3). For the 4-year sequential windows, the first version would start in 1932 with standard deviations calculated for 4-year sequential windows: 1932-1936, 1937-1941, etc…, the second version would start in 1933: 1933-1937, 1938-1942, etc… To determine if variability is increasing for each window size, we calculated the Theil-Sens slope and intercept. Ultimately, shorter windows had the most sequential standard deviation windows possible but the least number of unique versions. The 13-year window had only 5 or 6 different sequential standard deviation calculations but the most unique versions. To balance these two factors, we used 9-year windows for both analyses.

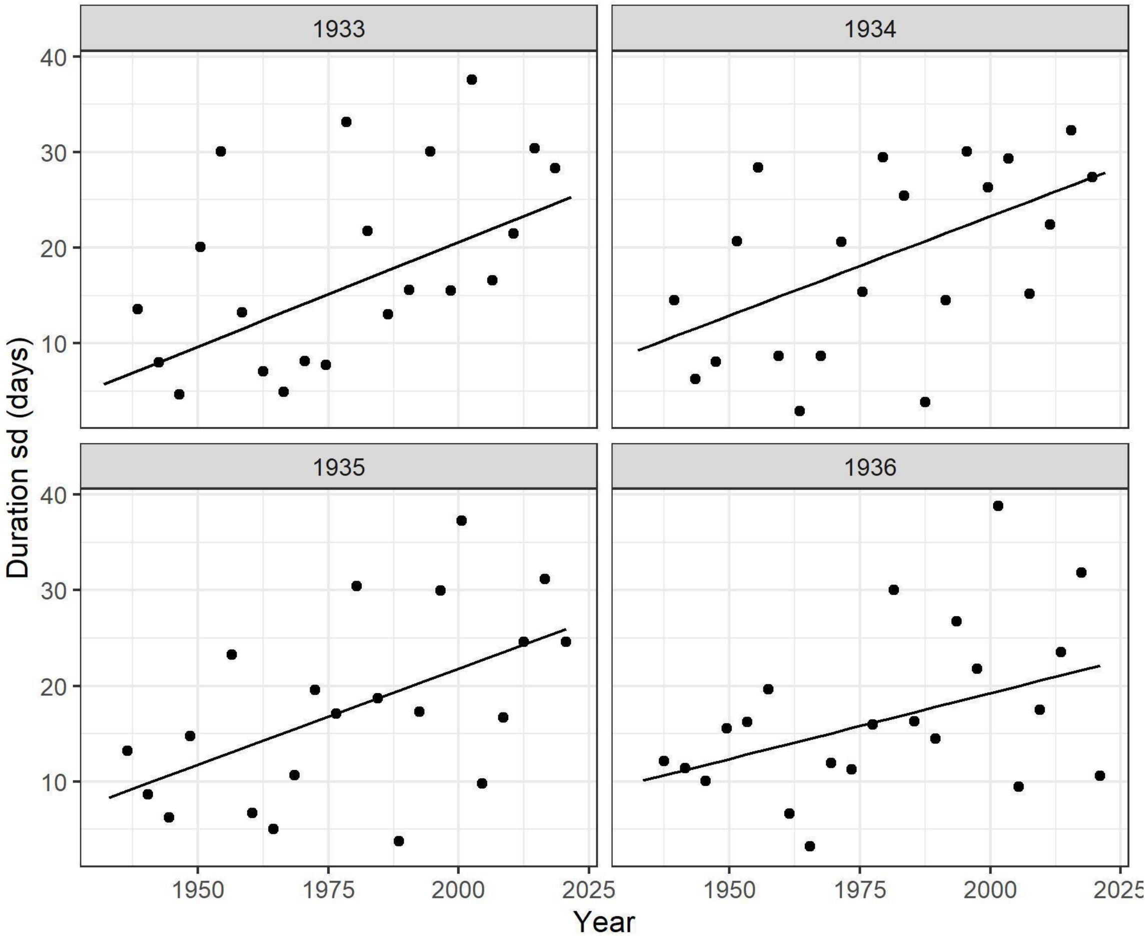

Figure S1. Standard deviations for all possible 4-year sequential windows where the year in each panel's title indicates the start year of the first segment. Additional sequential windows would overlap existing windows. The line indicates the Theil-Sen slope for each sequence of standard deviations.

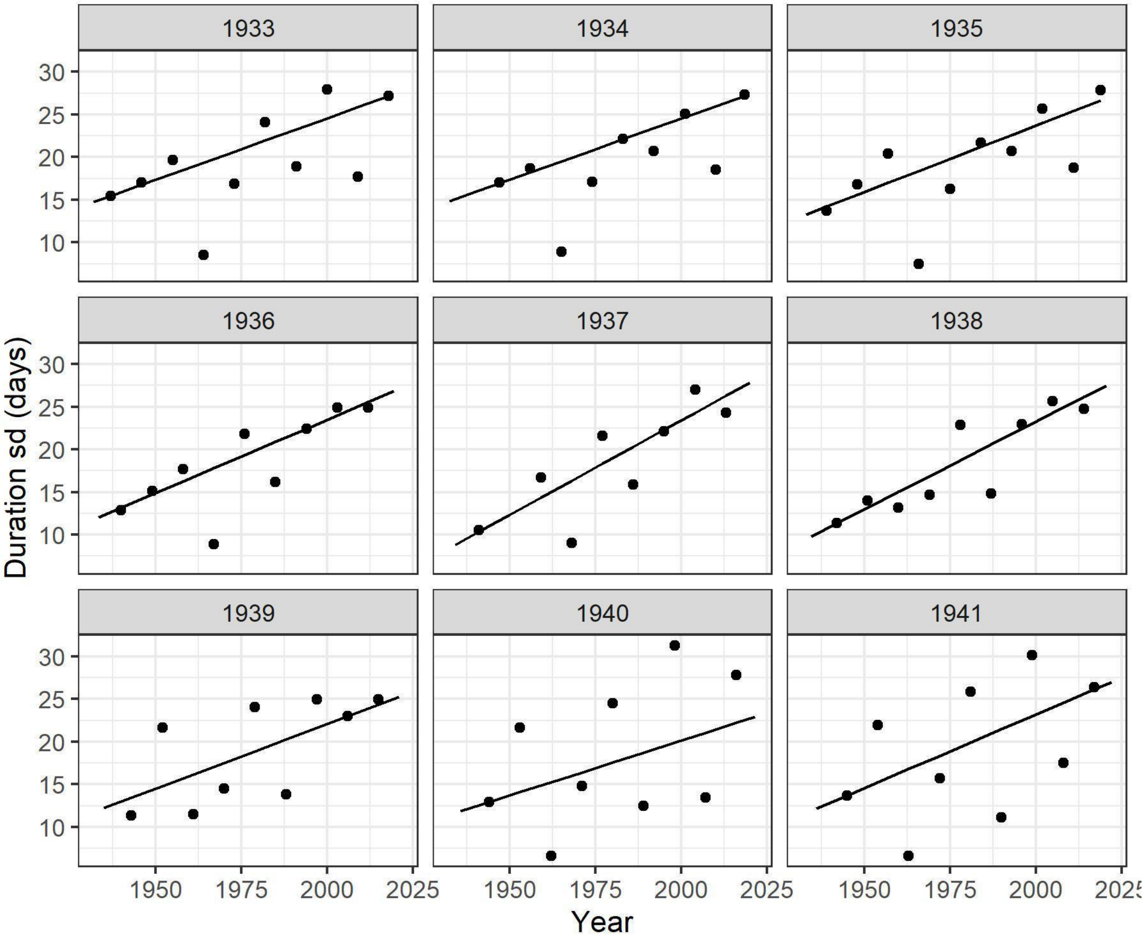

Figure S2. Standard deviations for all possible 9-year sequential windows where the year in each panel's title indicates the start year of the first segment. Additional sequential windows would overlap existing windows. The line indicates the Theil-Sen slope for each sequence of standard deviations.

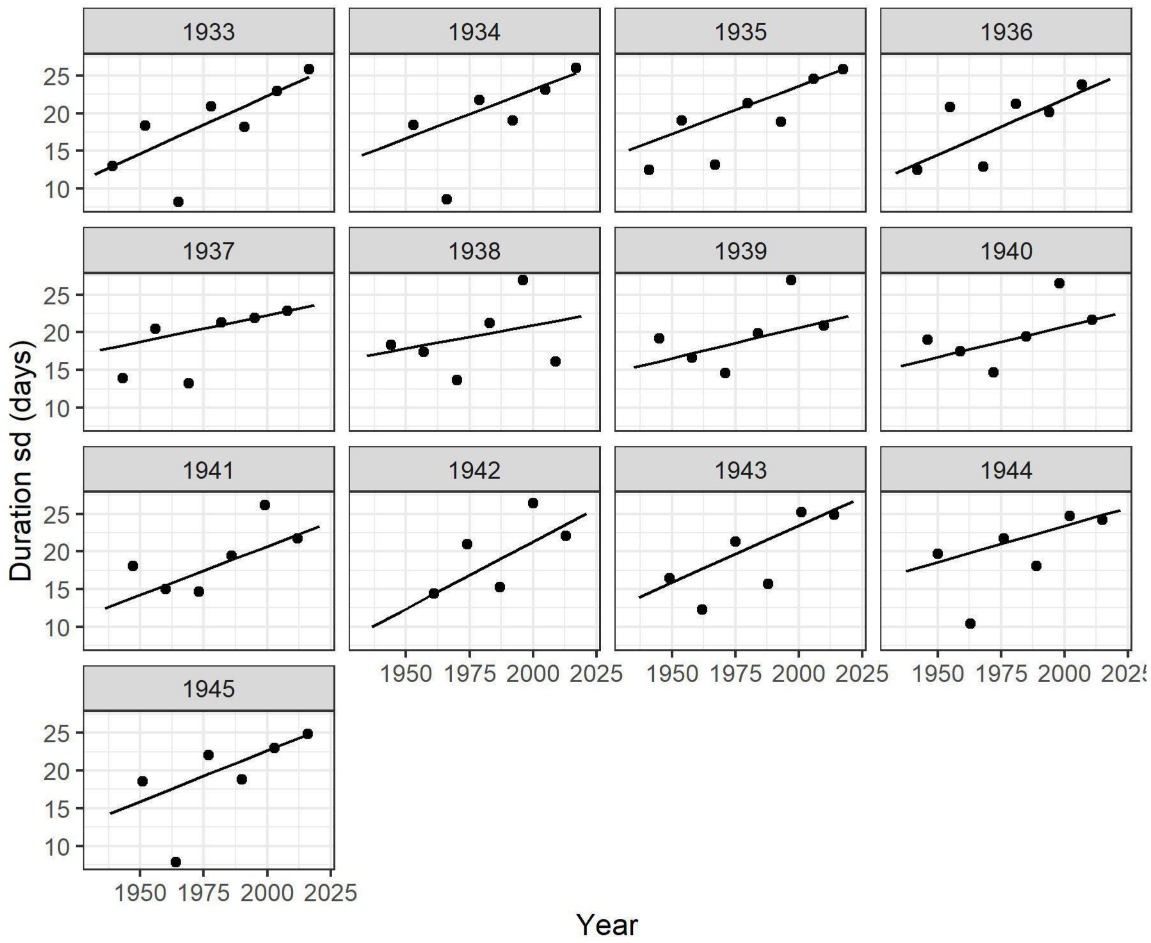

Figure S3. Standard deviations for all possible 13-year sequential windows where the year in each panel's title indicates the start year of the first segment. Additional sequential windows would overlap existing windows. The line indicates the Theil-Sen slope for each sequence of standard deviations.

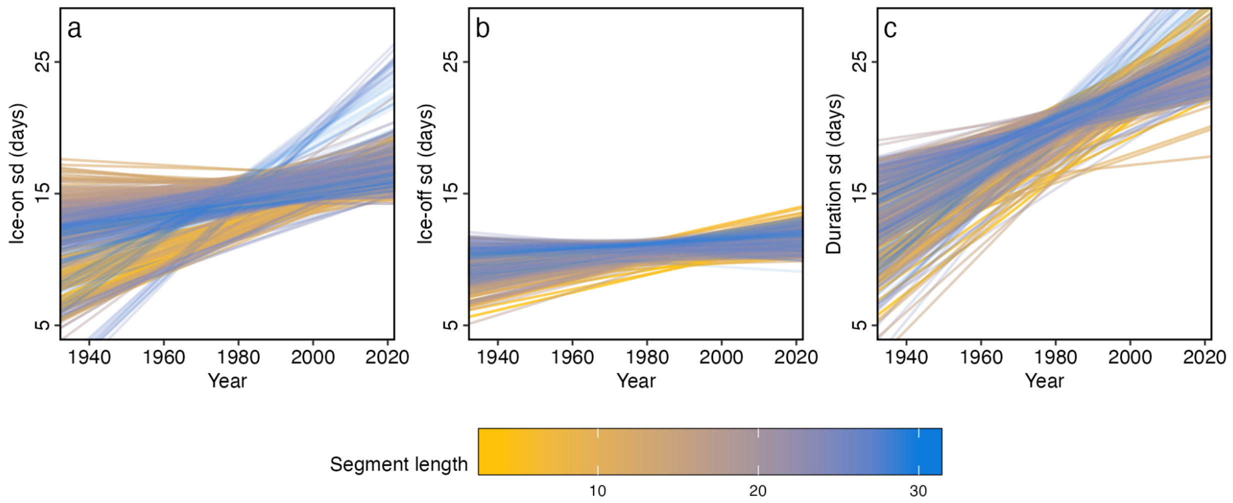

Figure S4. Theil-Sen slope fits for (a) ice-on standard deviation (sd), (b) ice-off sd, and (c)

duration sd compared across years of record (1931-2022). Sds were calculated from sequential windows of time across 4 to 30 years with color representing the sequential window length (segment length). For example, four year windows had four unique slopes (see Figure S1), 13-year sequential windows had 13 different slopes (see Figure S3), and 30 year windows had 30 different slopes. Overall, this resulted in 459 total best-fit lines in each panel.

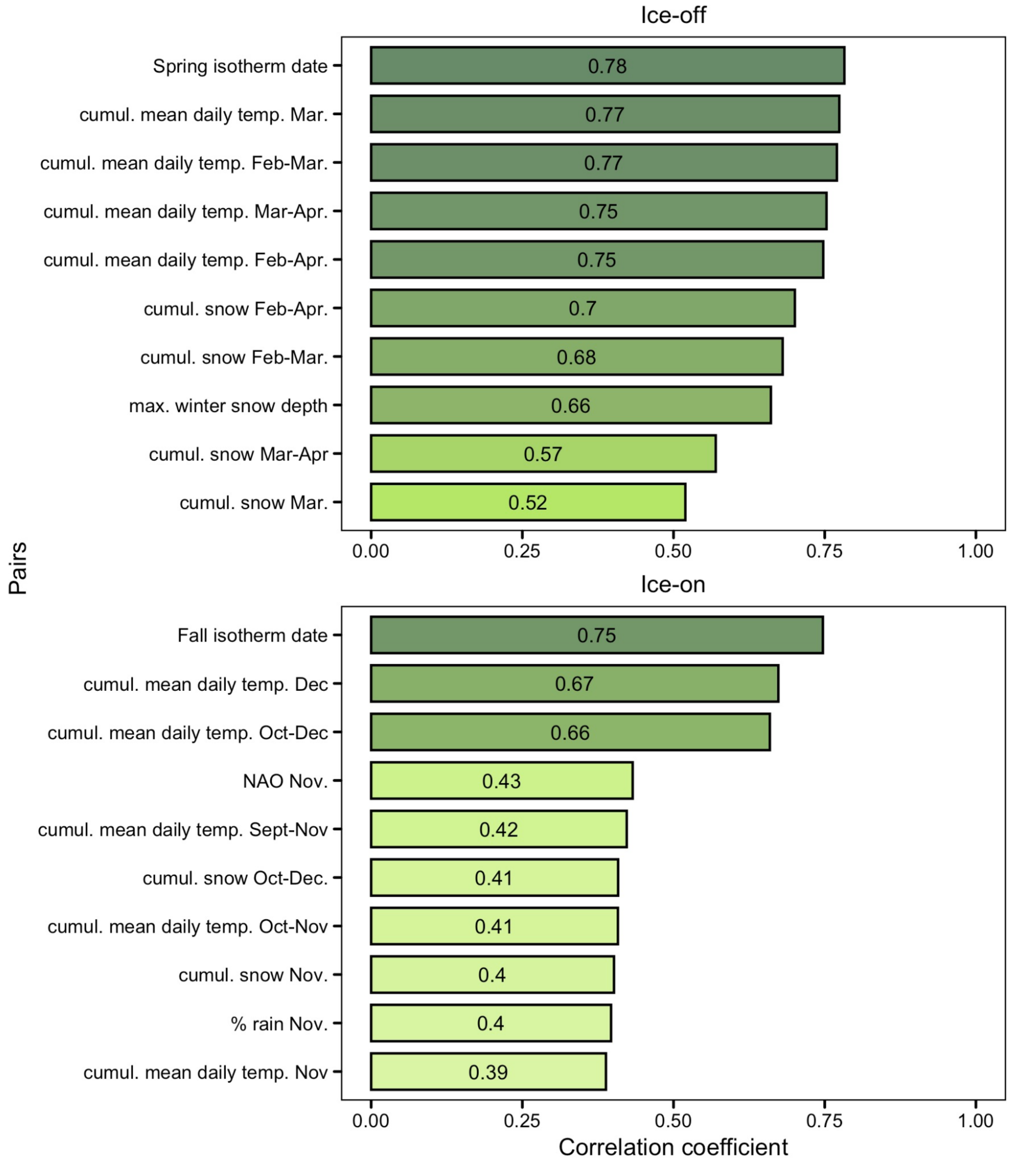

Figure S5. Pairwise correlation (Spearman's rho) between ice-off (top panel) and ice-on (bottom panel) and variation meteorological variables ranked in order from strongest to weakest. Prior to building the models, we tested for collinearity among the variables. For collinear pairs with correlation >|0.7|, we chose the variable with the higher correlation strength with ice-on or ice-off date.

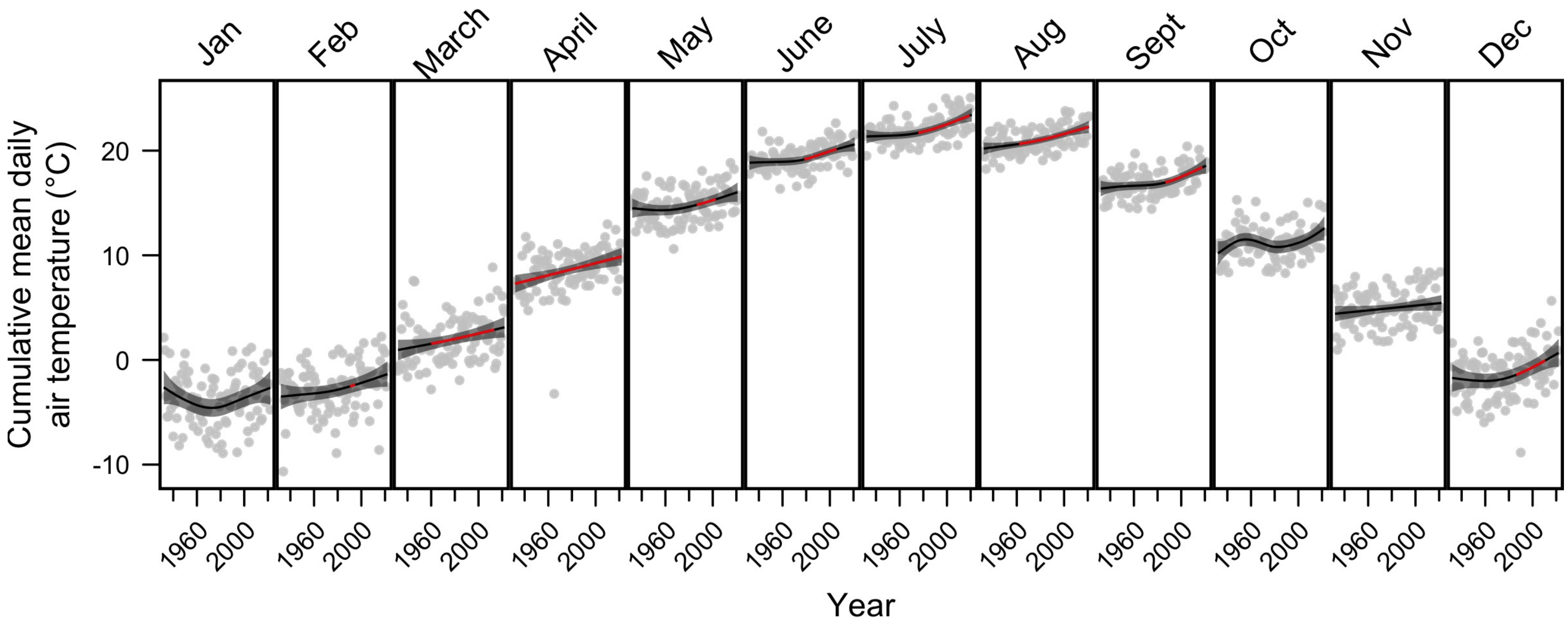

Figure S6. Cumulative mean daily air temperatures at Mohonk Lake between 1932 and 2022. Grey points are raw data and lines are a fitted trend. Shading around trend shows 95% confidence intervals. Red lines indicate periods of a significant temperature increase as indicated by the first derivative of the generalized additive model.

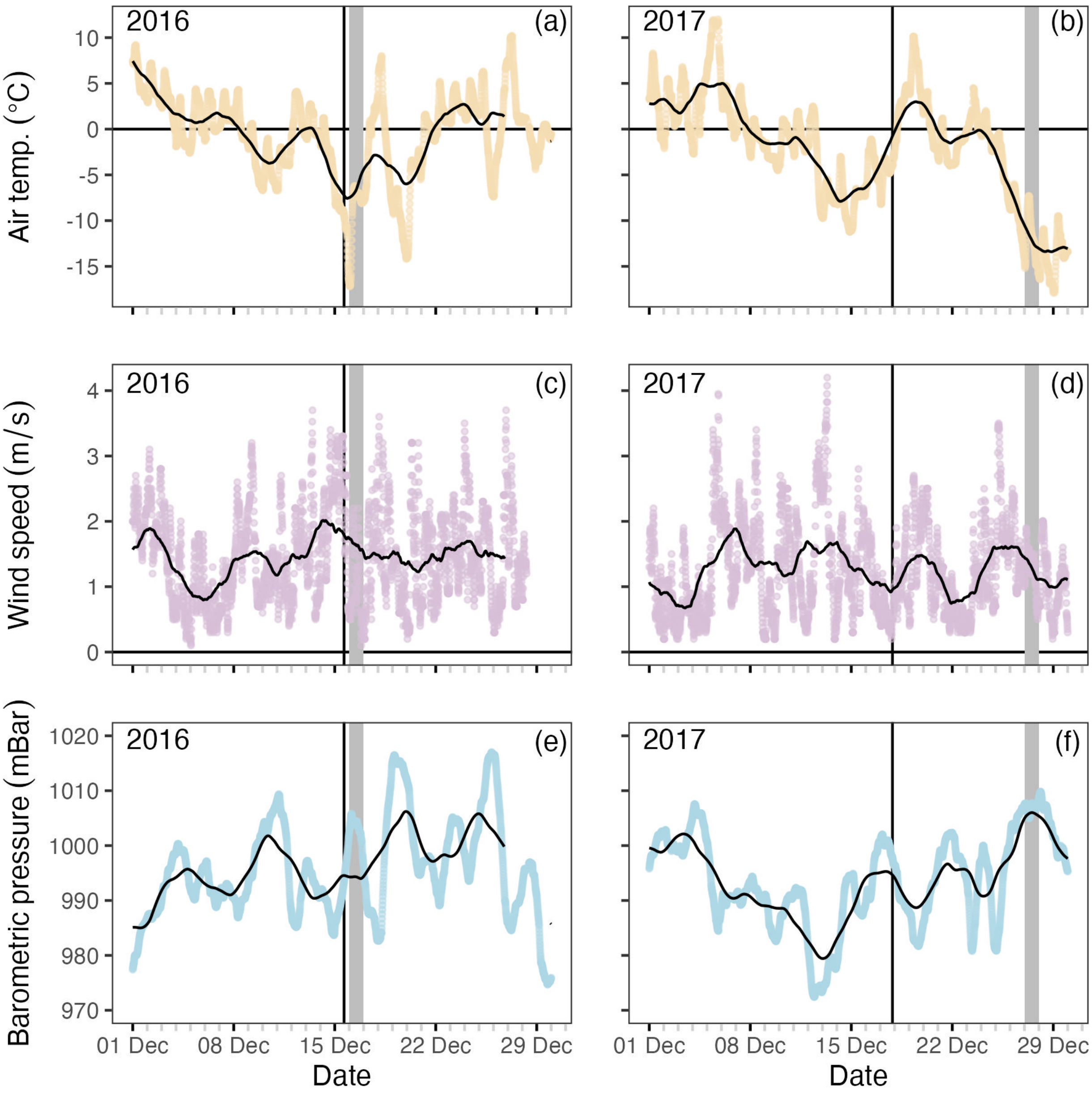

Figure S7. Meteorological data for the beginning of winter in 2016 (a, c, e) and 2017 (b, d, f) for air temperature (top row), wind speed (middle row), and barometric pressure (bottom row). The black line is the 3 day moving average. The vertical black line is ice-on identified by high-frequency temperature differences between 0 m and 9 m temperature sensors. The grey bar indicates the day (full 24-hour period) identified from visual assessment as the first day of the winter with 100% ice cover.

Table S1. List of top 3 GAMs fitted to ice-on and ice-off date. Model complexity (EDF; effective degrees of freedom) and AIC scores are shown for each model. The models highlighted in the text are ranked #1 and model summaries are contained in supplemental Table S2.

**Response**	**Predictors**	**EDF**	**AIC**	**Rank**	**% Dev. explained**
Ice on date	Fall isotherm date,<br>Cumulative mean daily temp. Nov.	3.6	461.8	1	67.7%
Ice on date	Fall isotherm date	2.0	478.9	2	56.4%
Ice on date	Cumulative mean daily air temp. Nov,<br>Cumulative mean daily air temp. Dec<br>November NAO index	4.0	546.7	3	57.9%
Ice off date (9)	Cumulative mean daily air temp. February,<br>Spring isotherm date,<br>Cumulative snowfall Feb.-Apr.,<br>Ice in day of year	6.9	461.5	1	81.2%
Ice off date (7)	Cumulative mean daily air temp. February,<br>Cumulative mean daily air temp. March,<br>Cumulative snowfall Feb.-Apr.<br>Ice in day of year	9.0	469.5	2	80.4%
Ice off date (8)	Cumulative mean daily temp. Feb.-Mar.,<br>Cumulative snowfall Feb.-Apr.,<br>Ice in day of year	8.2	473.3	3	78.8%

Table S2. Top ranking model summary for ice on date and ice off date.

		Estimate	Est. df.	Ref. df	Chi.sq	F	p-value
**Ice duration**	Intercept	97.6					
	Global temperature anomaly		1.0	1.0	11.4	-	< 0.001
	NAO index Nov.		1.0	1.1	3.2	-	0.082
	NAO index Dec.		1.0	1.0	3.7	-	0.054
**Ice-on date**	Intercept	4.4					
	Fall isotherm date		1.0	1.0	-	110.4	< 0.001
	Cumulative mean daily temp. Nov.		1.6	1.9	-	11.8	< 0.001
**Ice-off date**	Intercept	95.7					
	Cumulative mean daily temp. Feb.		1.0	1.0	-	16.9	< 0.001
	Spring isotherm date		1.0	1.0	-	74.9	< 0.001
	Cumulative snow Feb-Apr.		2.8	3.6	-	8.4	< 0.001
	Ice-on day of year		1.0	1.0	-	1.8	0.18

Table S3. Sens slopes on all the computed climatic variables for Mohonk Lake. We computed Sens slopes for all monthly and seasonal ENSO and NAO variables, but all Sens slope p-values were > 0.05 and therefore not printed in this table.
*** p < 0.001; ** p < 0.01; * p < 0.05.

**Climatic variable**	**p-value**	**Slope**	**Intercept**	**z-statistic**
Global temp. anomaly (°C)	<0.001***	0.0112	-21.8	9.93
Date of maximum snowfall	0.8183	0.167	-186	0.23
Maximum snow depth (mm)	0.7162	-2.21	4870-	-0.363
Sept. cumulative snow (mm)	1	0	0	0
Sept.-Nov. cumulative rain (mm)	0.114	0.865	-1400	1.58
Sept.-Nov. number of days mean daily air T below zero	0.0558	-0.0217	47.2	-1.91
Sept.-Nov. number of days min daily air T below zero	0.18	-0.0294	71.4	-1.33
Sept.-Oct. number of days min daily air T below zero	0.2657	0	1	-1.11
Sept.-Nov. cumulative mean daily air temp. (°C)	0.0013**	1.35	-1650	3.23
Sept.-Nov. cumulative snow (mm)	0.261	-0.334	715	-1.12
Sept.-Oct. cumulative mean daily air temp. (°C)	0.0007***	0.978	-1070	3.39
Sept.-Oct. cumulative rain (mm)	0.0347*	0.975	-1730	2.11
Sept.-Oct. cumulative snow (mm)	0.9583	0	0	-0.05
Sept. cumulative mean daily air temp. (°C)	0.0001***	0.644	-760	3.9

Climatic variable	p-value	Slope	Intercept	z-statistic
Sept. cumulative rain (mm)	0.221	0.371	-631	1.22
Oct cumulative mean daily air temp. (°C)	0.0984	0.323	-294	1.65
Oct.-Dec cumulative mean daily air temp. (°C)	0.0098**	1.32	-2170	2.58
Oct.-Dec. cumulative rain (mm)	0.0362*	0.957	-1590	2.09
Oct.-Dec. cumulative snow (mm)	0.805	-0.526	1460	0.247
Oct.-Dec. number of days mean daily air T below zero	0.0058**	-0.087	196	-2.76
Oct.-Dec. number of days min daily air T below zero	0.097	-0.0496	138	-1.66
Oct.-Nov cumulative mean daily air temp. (°C)	0.0378*	0.677	-843	2.08
Oct.-Nov. cumulative rain (mm)	0.084	0.578	-938	1.73
Oct.-Nov. number of days mean daily air T below zero	0.0515	-0.0204	44.6	-1.95
Oct.-Nov. number of days min daily air T below zero	0.1649	-0.0294	71.5	-1.39
Oct. % of precipitation as rain	0.9397	0	100	-0.07
Oct. cumulative rain (mm)	0.0126*	0.615	-1.12e+03	2.5
Oct. cumulative snow (mm)	0.9934	0	0	-0.0083
Oct. number of days min daily air T below zero	0.0265*	0	1	-2.22
Oct.-Nov. cumulative snow (mm)	0.1598	-0.406	852	-1.41

Climatic variable	p-value	Slope	Intercept	z-statistic
Nov cumulative mean daily air temp. (°C)	0.1664	0.331	-510	1.38
Nov. % of precipitation as rain	0.0362*	0.184	-293	2.09
Nov. cumulative rain (mm)	0.715	-0.0768	251	-0.365
Nov. cumulative snow (mm)	0.0473	-0.305	651	-1.98
Nov. number of days mean daily air T below zero	0.0608	-0.0184	40.6	-1.88
Nov. number of days min daily air T below zero	0.3148	-0.0177	47.2	-1.01
Dec. % of precipitation as rain	0.7778	0.0176	-9.63	0.282
Dec. cumulative rain (mm)	0.0143*	0.459	-804	2.45
Dec. cumulative snow (mm)	0.3613	0.901	-1.53e+03	0.913
Fall isotherm date	0.0292*	0.214	-330	2.18
Dec. number of days mean daily air T below zero	0.005*	-0.0714	159	-2.81
Dec. number of days min daily air T below zero	0.0725	-0.0303	85.5	-1.8
Dec. cumulative mean daily air temp. (°C)	0.0024**	0.844	-1.7e+03	3.04
Jan cumulative rain (mm)	0.4315	0.148	-209	0.787
Jan-Mar cumulative mean daily air temp. (°C)	0.0109*	1.54	-3.19e+03	2.55
Jan-Mar. cumulative rain (mm)	0.333=91	0.407	-535	0.956

**Climatic variable**	**p-value**	**Slope**	**Intercept**	**z-statistic**
Jan-Mar. cumulative snow (mm)	0.7551	-0.788	2.56e+03	-0.312
Jan. % of precipitation as rain	0.5261	0.0418	61.5	0.634
Jan. cumulative mean daily air temp. (°C)	0.5817	0.228	-571	0.551
Jan. cumulative snow (mm)	0.6469	-0.473	1.29e+03	-0.458
Jan. number of days mean daily air T below zero	0.5515	0	24	-0.596
Jan. number of days min daily air T below zero	0.1582	0	29	-1.41
Feb cumulative mean daily air temp. (°C)	0.018*	0.639	-1.34e+03	2.37
Feb-Apr. cumulative mean daily air temp. (°C)	<0.0001***	2.59	-4.88e+03	4.26
Feb-Apr. cumulative rain (mm)	0.6807	0.119	32.8	0.441
Feb-Apr. cumulative snow (mm)	0.6233	-1.08	2.87e+03	-0.491
Feb-Mar cumulative mean daily air temp. (°C)	0.001**	1.59	-3.17e+03	3.3
Feb-Mar. cumulative rain (mm)	0.7677	0.08	-20.1	0.295
Feb-Mar. cumulative snow (mm)	0.9154	-0.159	1.01e+03	-0.106
Feb-Mar. number of days mean daily air T above zero	0.003**	0.0945	-158	2.97
Feb-Mar. number of days min daily air T above zero	0.0015***	0.0789	-145	3.18

Climatic variable	p-value	Slope	Intercept	z-statistic
Feb. % of precipitation as rain	0.628	0.0204	-22.2	0.484
Feb. cumulative rain (mm)	0.9102	0.0136	48.2	0.113
Feb. cumulative snow (mm)	0.5748	-0.564	1.45e+03	-0.561
Feb. number of days mean daily air T below zero	0.0823	-0.0348	88.5	-1.74
Feb. number of days min daily air T below zero	0.0098**	-0.025	75.3	-2.58
Mar-Apr cumulative mean daily air temp. (°C)	0.0001***	1.79	-3.21e+03	3.99
Mar-Apr. cumulative rain (mm)	0.548	0.196	-197	0.601
Mar-Apr. cumulative snow (mm)	0.18	-1.65	3.61e+03	-1.34
Mar-Apr. number of days mean daily air T above zero	0.0039**	0.0625	-73.9	2.98
Mar-Apr. number of days min daily air T above zero	0.0026***	0.0816	-129	3.01
Mar. % of precipitation as rain	0.4984	0.0429	-59.1	0.667
Spring isotherm date	0.0008***	-0.144	476	-3.36
Mar. cumulative mean daily air temp. (°C)	0.0024**	0.935	-1.79e+03	3.04
Mar. cumulative rain (mm)	0.4219	0.152	-204	0.803
Mar. cumulative snow (mm)	0.7324	-0.286	849	-0.342
Mar. number of days mean daily air T above zero	0.0045**	0.0603	-99.3	2.84

Climatic variable	p-value	Slope	Intercept	z-statistic
Mar. number of days mean daily air T below zero	0.0045**	-0.0603	130	-2.84
Mar. number of days min daily air T above zero	0.0232**	0.0463	-83.2	2.27
Mar. number of days min daily air T below zero	0.0232**	-0.0463	114	-2.27
Apr. % of precipitation as rain	0.0654	0	85.9	1.84
Apr. cumulative mean daily air temp. (°C)	0.0001***	0.978	-1.67e+03	4.04
Apr. cumulative rain (mm)	0.926	-0.0272	158	-0.0929
Apr. cumulative snow (mm)	0.0659	0	12.7	-1.84
Apr. number of days mean daily air T below zero	0.1521	0	0	-1.43
Apr. number of days min daily air T below zero	0.0044**	-0.04	84.8	-2.85

Table S4. Structural equation model fits. Relationship indicates the type of relationship (covariance or regression) for each variable with the variable (indented) below. For regression, variables indicate response variable (non-indented) and predictor variable or intercept (indented). For each relationship, an estimate of fit (Estimate), standard error of the estimate (Estimate SE), test-statistic (Z statistic) and p-value are included.

**Variables**	**Relationship**	**Estimate**	**Estimate SE**	**Z statistic**	**p-value**
Ice-on date					
Fall mixed period (d)	Covariance	0.84	0.26	3.16	*0.002*
Under ice shallow (°C)					
Ice-on date	Regression	-0.23	0.19	-1.20	0.229
Intercept	Regression	0.09	0.19	0.47	0.637
Under ice deep (°C)					
Ice-on date	Regression	-0.43	0.17	-2.52	*0.012*
Intercept	Regression	0.10	0.17	0.60	0.549
Under ice deep (°C)					
Under ice shallow (°C)	Covariance	0.70	0.22	3.26	*0.001*
Density Δ (kg m^{-3})					
Under ice deep (°C)	Regression	-0.87	0.36	-2.42	*0.016*
Under ice shallow (°C)	Regression	1.27	0.35	3.58	*<0.001*
Intercept	Regression	0.01	0.16	0.03	0.974
Heat content (MJ)					
Under ice deep (°C)	Regression	0.43	0.03	14.52	*<0.001*
Under ice shallow (°C)	Regression	0.61	0.03	19.13	*<0.001*
Density Δ (kg m^{-3})	Regression	-0.05	0.01	-3.09	*0.002*
Intercept	Regression	0.01	0.01	0.59	0.557
Ice-off date					
Density Δ (kg m^{-3})	Regression	-0.12	0.23	-0.50	0.614
Under ice shallow (°C)	Regression	0.00	1.68	0.00	0.999
Under ice deep (°C)	Regression	0.87	1.20	0.73	0.466
Heat content (MJ)	Regression	-0.81	2.67	-0.30	0.762
Intercept	Regression	0.01	0.19	0.06	0.955
Ice-off date					
Spring mixed period (d)	Covariance	-0.49	0.20	-2.43	*0.015*
